# Enhancing Thermal Insulation of EPDM Ablators via Constructing Alternating Planar Architectures

**DOI:** 10.3390/polym14081570

**Published:** 2022-04-12

**Authors:** Hongjian Qu, Le Wang, Kun Hui, Cheng Bian, Hongyan Li, Yiwen Guan, Tao Luan, Ning Yan

**Affiliations:** Xi’an Modern Chemistry Research Institute, Xi’an 710065, China; hjq2042021@163.com (H.Q.); wangle_204@163.com (L.W.); hk_0327@163.com (K.H.); biancheng159@126.com (C.B.); gyw@mail.nwpu.edu.cn (Y.G.); yimubath@163.com (T.L.)

**Keywords:** ablation, thermal insulation, multilayer, EPDM, porous

## Abstract

Ethylene–propylene–diene monomer (EPDM) composites were usually enhanced with ablative additives to protect solid rocket motor (SRMs) casings. However, the poor thermal insulation caused by the high thermal conductive ablative fillers can lead to rocket motor failure. Herein, the novel EPDM composites containing alternating layers of ablative EPDM (AM) and heat-insulated EPDM (HM) were prepared through layer-multiplying extrusion. Compared with conventional EPDM ablative material, the multilayer composites showed enhanced thermal insulation and mechanical properties that could be further improved by tuning the number of layers. The ablation and thermal insulation properties possessing in AM and HM layers could be combined by forced assembly during co-extrusion, and the alternating multilayer composite was capable of showing the effect of each component. In particular, compared with AM, the maximum back-face temperature with 40 alternating layers of AM/HM decreased from 96.2 °C to 75.6 °C during oxyacetylene test, while the good ablation properties were preserved in the AM component. This significant improvement was attributed to the planar orientation and densification of ablative additives, and the interruption of conductive pathways in the through-plane direction of AM/HM alternating laminate. The anisotropic EPDM composites featuring mechanical robustness, good ablative resistance and thermal insulation suggest considerable potential application in the aerospace industry.

## 1. Introduction

Polymeric ablation materials (PAMs) represent one of the most important parts of thermal protection system used in solid rocket motors (SRMs) [1]. Compared with conventional ablation materials such as C/C composites, C/phenolic composites and ceramic composites [2,3], PAMs have more inborn benefits, such as corrosion resistance, low density, good mechanical properties and thermal insulation capabilities. SRMs generate severe heat during operation. The existence of PAMs ensures reliable bonding and flexible adaptability between the propellant and motor case [4], and protects the structural integrity of the SRMs in severe heating flow. Among various PAMs, EPDM is one of the most ideal substrates due to better anti-aging performance and lower density (0.80–0.87 g/cm^3^) [5,6,7,8,9].

EPDM-based ablation materials are a type of carbonized composite that can generate a char layer during ablation [10,11], endowing the virgin EPDM composites with a dense and integral shield. As the char layer resists the chemical erosion and mechanical denudation of the products of combustion gases, the thermal stability and strength of the char layer are key factors affecting the ablative performance [12,13]. Hence, various ablative additives, such as fibers [14,15,16,17] and ceramic fillers [18,19,20], have been extensively used in PAMs to increase the integrity and strength, and decrease the oxidation rate of the char layer. Among these reinforcements, the addition of fibers facilitates the formation of an embedded skeleton structure, which allows the enhancement of polymeric matrices, and improves the dimensional and thermal stability once the polymeric matrix is charred, resulting in a decrease in the erosion rate and an increase in the char adhesion on the virgin substrate [21,22,23]. In addition, the presence of ceramic fillers is beneficial for densifying the char layer, because the ceramic additives can melt and convert into a viscous liquid in a hyperthermal atmosphere. The impregnation of viscous liquid can promote the sintering and ceramization of the char layer under the carbothermal reaction, allowing an enhancement of the hardness and strength of the char layer [24,25].

Regardless of these benefits, the addition of large amounts of fillers usually causes an increase in both material density and thermal conductivity, and worsens the robustness of PAMs [26]. Moreover, the ceramic products generated during ablation show a very high density and thermal conductivity, which tend to accelerate the pyrolysis of the inner matrix, and are harmful for the practical use of PAMs as a liner in SRMs [27]. In particular, the addition of hybrid fibers in the ablation materials form fibrous conductive networks, which facilitate the formation of numerous hot spots and accelerate heat diffusion. The temperature rise weakens the shell strength and imposes an adverse effect on the security of SRMs during the operation.

Typically, ideal PAMs should have a low density and be mechanically robust to ensure a high payload of the rocket, and should have a low thermal conductivity to achieve excellent thermal insulation. More importantly, the char layer originating from the ablation of PAMs should have low thermal conductivity and high strength to resist the heat diffusion and physicochemical erosion of combustion gases [28]. However, endowing EPDM composites with versatility was very challenging because functional additives were mixed randomly through conventional compounding methods, and only isotropic dispersion in the matrix can be achieved, leading to limited reinforcement of the comprehensive properties. Therefore, tailoring the morphologies of each component provides a potential route to achieve desired multifunctional properties.

The multilayer structure combining the properties of each component was considered to be a good choice for achieving balanced ablation and thermal insulation performances for the EPDM composites. Ahmed et al. [29] prepared the multilayered PAMs consisting of alternating layers of EPDM/carbon fiber and EPDM/Kevlar fiber components through a conventional compression process. Compared with the randomly mixing counterpart, the thermal conductivity decreased by 20%, while the ablation property was preserved, indicating that each component of the multilayer composite is capable of endowing benefits to the ablation and thermal insulation.

Compared to the routine method, the emerging co-extrusion technology makes the preparation of multilayer structures more controllable and efficient by layer multiplying elements (LMEs) [30,31]. Especially, the use of LME offered an attractive approach to orient the anisotropic fillers with high aspect ratio, such as fibers and nanoplatelets, in the layer plane, which provided a potential route to achieve unique dispersion of high-volume fraction and in-plane alignment of anisotropic additives [32]. Therefore, the morphologies of each component could be tailored, which affords optimum functional properties. In this work, the EPDM composites consisting of alternating layers of ablative EPDM (AM) and heat-insulated EPDM (HM) were fabricated through layer-multiplying extrusion. The layer morphologies, anisotropic thermal conductivity and mechanical properties were systematically investigated, and the influence of the multilayer structure on the ablation and heat-insulation performances was studied to offer guidance for the preparation of high-performance PAMs.

## 2. Experimental

### 2.1. Materials

EPDM (Type 4045M of Mitsui, Tokyo, Japan) with 44.5 wt% ethylene content and 7.5% ethylidene 2-norbornene content was purchased as matrix material. Liquid EPDM was provided by Shaanxi Hengzhi Fine Chemicals Co., Ltd. (Xi’an, China). Aramid fiber (AF) with diameter about 20 μm and length of 6–8 mm was purchased from Mongolia Hexi Aerospace Technology Development Co., Ltd. (Inner Mongolia, China) Carbon fiber(CF) with diameter about 7 μm and length of 1 mm was purchased from Shanghai Dongfang Technology Development Co., Ltd. (Shanghai, China) Borosilicate microspheres and boron phenolic were provided by Shaanxi Taihang Fire Resistant Polymer Co., Ltd. (Xi’an, China) Zinc oxide and stearic acid were provided by Chengdu Kelong Chemical Co., Ltd. (Chengdu, China) Diphenylguanidine (D) and dibenzothiazole disulfide (DM) were purchased from Sanmen Haichuan Chemical Co., Ltd. (Taizhou, China), and zinc diethyldithiocarbamate (EZ) was purchased from Weilin New Material Technology Co., Ltd. (Puyang, China) Silicon dioxide and sulfur were supplied by Xilong Science Co., Ltd. (Xi’an, China) and Shanghai Yuejiang Titanium White Chemical Products Co., Ltd. (Shanghai, China), respectively. All chemicals were used without further purification.

### 2.2. Preparation of Alternating Multilayer Composites

#### 2.2.1. Preparation of Ablative and Heat—Insulation Components

To prepare the ablative EPDM composite, first, EPDM was blended with aramid fiber in a twin-roll mill with a nip gap of 0.5 mm at room temperature, and the compound was extruded several times to ensure a good distribution of fibers. Then, other ingredients such as silica and vulcanizing agents were sequentially added until uniform mixing was achieved. After that, brittle CFs were added at a larger nip gap to mitigate their fracture. The obtained ablative compound was abbreviated as AM. The heat-insulated EPDM composite was prepared by following the same procedure without the addition of fibers. In brief, glass microspheres were firstly mixed with liquid EPDM to prevent them from breaking. Then, the resulting compound was blended with EPDM matrix, silica and vulcanizing agents. The resultant was designated as HM, where the presence of porous medium can reduce thermal conductivity. The formulations of AM and HM are shown in Table S1.

#### 2.2.2. Preparation of the Multilayer EPDM Composites

**Figure 1:**
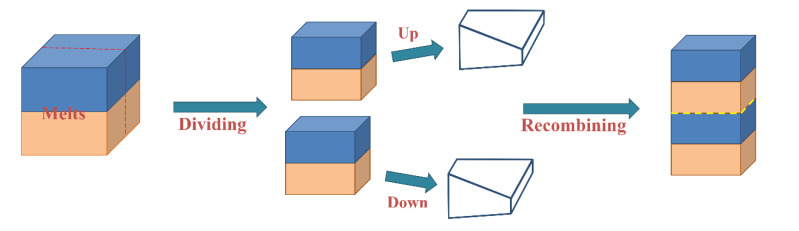
Schematic illustration of layer multiplying co-extrusion.

**Figure 2:**
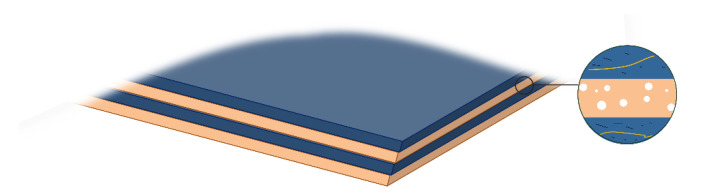
Schematic diagram of the multilayer EPDM composite.

The multilayer EPDM composites were prepared using a co-extrusion system, as schematically illustrated in Figure 1. The screw of the rubber extruder (SJ-40 × 12) with a length/diameter ratio (L/d) of 12:1 was produced by Zhangjiagang Lianjiang Machinery Co., Ltd. (Zhangjiagang, China) The temperature of all extrusion zones was maintained at 80 °C, and the AM and HM components were extruded from two extruders, respectively, merging into two-layer melt. Then, the melt passed through a group of layer multiplying elements (LMEs), where the melt was averagely divided into two sections, and the recombination led to the doubling of layer numbers. Typically, the assembly of *n* LMEs enables the fabrication of materials with 2^*n*+1^ layers. In this work, 0, 1, 2, and 3 LMEs were used to fabricate EPDM composites with 2, 4, 8, and 16 layers, respectively. The thickness of all extruded sheets was maintained at 2 mm, and the extrusion ratio of AM and HM was kept at 1:1. The compounds were molded at 150 °C, and the multilayered EPDM composites with different layers were abbreviated as (AM/HM)_2_, (AM/HM)_4_, (AM/HM)_8_ and (AM/HM)_16_, respectively. Considering that the thickness of the specimen in ablation and compression tests is 10 mm, five sheets were piled up and molded, and the resultants were designated as (AM/HM)_10_, (AM/HM)_20_, (AM/HM)_40_ and (AM/HM)_80_, respectively. For comparison, the AM and HM composites were also prepared by compressive molding at 150 °C, respectively. The schematic diagram of the multilayer composite is shown in Figure 2, where AM is represented in blue and HM with the porous medium is represented in orange.

### 2.3. Characterization 

#### 2.3.1. Thermal Stability and Thermal Conductivity Test 

Thermogravimetric analysis (TGA) of AM, HM and AM/HM composites was performed in the NETZSCH STA 449F3(NETZSCH-Gerätebau GmbH, Selb, Germany) instrument. The test sample is consistent with the structure of the alternating multilayer structure material. The sample was tested from 30 °C to 800 °C at a heating rate of 5 °C/min in the atmosphere of nitrogen. 

The thermal conductivity of the sample at room temperature was tested by the transient flat heat source method instrument (Hot Disk TPS2500S, Hot Disk AB, Gothenburg, Sweden). Thermal conductivity of the EPDM composites in both in-plane and through-plane directions was measured.

#### 2.3.2. Mechanical Properties Test

The mechanical properties of the EPDM composites were measured according to the China standard GB T528. The tensile properties are the average value of five specimens. The compressive properties of the material are tested on a cylindrical-shaped specimen with a diameter of 29.5 mm and a thickness of 12.5 mm. The samples were compressed to 40% deformation at room temperature, the strain rate was 10 mm/min and four compression cycles were repeated. The compressive modulus was calculated based on the fourth compression curve, and the average values of the three specimens were considered as the compressive properties.

#### 2.3.3. Ablation Test

**Figure 3:**
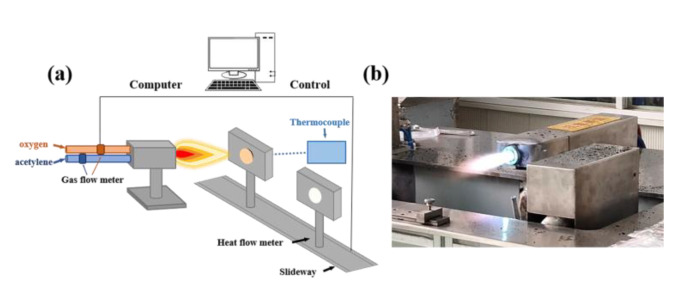
Schematic diagram (**a**) and realistic picture (**b**) of the oxyacetylene ablation instrument.

In this work, the ablation performance of the EPDM composites was evaluated using the oxyacetylene ablation instrument (Figure 3). The oxygen-acetylene gas flow rate was set at the range of 1512 L/h to 1116 L/h. The distance of the nozzle tip to the specimen was set as 10 mm, and the heat flux was tuned at 4475 kW/m^2^. The dimension of the specimen used for the ablation test was Φ30 × 10 mm. The oxyacetylene flame burnt vertically on the center of the specimen, with the ablation process lasting for 20 s. The linear ablation rate (*R_l_*), charring rate (*R_c_*) and mass ablation rate (*R_m_*) were calculated by the following formulas [13,28]:

(1) $$R_{l}=\frac{L_{1}-L_{2}}{t}$$

(2) $$R_{c}=\frac{L_{1}-L_{2}}{t}$$

(3) $$R_{m}=\frac{m_{1}-m_{2}}{t}$$

where *L*_1_ and *L*_2_ are the thicknesses of the specimen before and after ablation, respectively; *L*_2_ is the thickness of the specimen without char layer; *m*_1_ and *m*_2_ are the masses of the specimen before and after ablation, respectively; and t is the ablating time (s). In addition, a K-type thermocouple was fixed on the back face of the specimen to record the maximum back-face temperature (T_b_).

#### 2.3.4. Morphology Observation

An optical microscope (GP-304K (Kunshan Gaopin Precision Instrument Co., Ltd., Kunshan, China)) was used to observe the macroscopic morphology of the multilayer structure. The scanning electron microscope (SEM, Quanta 600F, FEI, Hillsboro, OR, USA) was used to observe the cross-sectional structure and filler distribution. In particular, the fiber distribution in the multilayered specimen along and vertical to the extrusion plane was observed. 

The macroscopic morphologies of the specimen after ablation were observed by optical microscopy. Moreover, the surface and cross-sectional morphologies of the char layer after ablation were observed by SEM. Energy-Dispersive X-ray Spectroscopy (EDS) was used to identify the elements at different positions of char layers.

## 3. Results and Discussion

### 3.1. Morphological Characterization 

**Figure 4:**
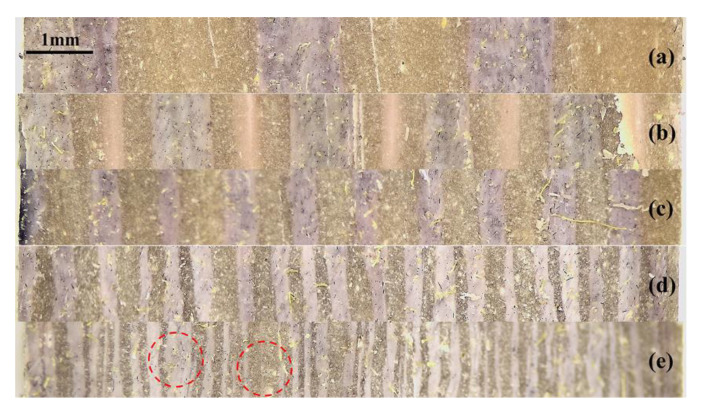
Optical microscopy image of the multilayer EPDM composites with different layers ((AM/HM)_6_: (**a**); (AM/HM)_10_: (**b**); (AM/HM)_20_: (**c**); (AM/HM)_40_: (**d**); (AM/HM)_80_: (**e**)).

**Figure 5:**
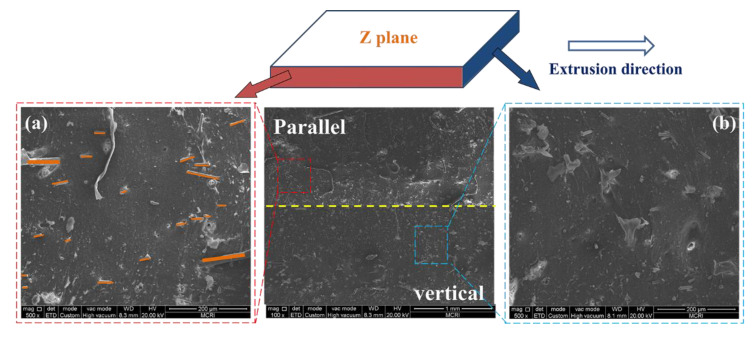
SEM images of the section of (AM/HM)_20_: parallel to the extrusion direction (**a**); perpendicular to the extrusion direction (**b**).

Figure 4 shows optical images of the multilayer EPDM composites with different layer numbers. The cross-sectional morphologies parallel to the extrusion direction shows a well-defined laminating structure, wherein the AM and HM components were alternatively distributed. Notably, the existence of strong multistage shear force during co-extrusion oriented the fibers in AM parallel to the extrusion direction (Figure 5a), whereas the filler dispersion perpendicular to the extrusion direction showed significant difference, as shown in Figure 5b. The size of microspheres calculated using the Nano Measure software showed a uniform dispersion at about 18 μm (the inset of Figure S1b), which is smaller than the initial size. The fibers and microspheres were confined in their parent matrices without penetrating into the adjacent layers. 

**Figure 6:**
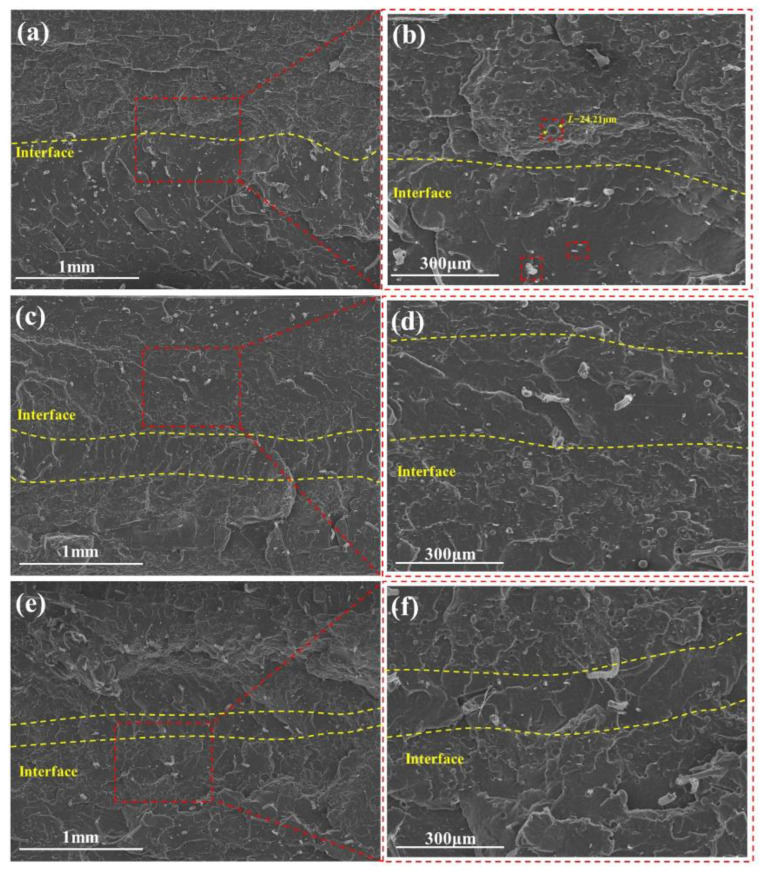
Cross-sectional SEM images of the multilayer EPDM composites: (**a**) (AM/HM)_10_, (**c**) (AM/HM)_40_, (**e**) (AM/HM)_80_; (**b**,**d**,**f**) are the partial magnification of (**a**,**c**,**e**), respectively.

The cross-sectional SEM images the multilayer EPDM composites with different layer numbers are shown Figure 6. The ablative EPDM (AM) and heat-insulated EPDM (HM) showed alternative distribution, the HM has a porous structure formed by microspheres, and the layered structure of the two components can be identified from the two different colors shown in Figure 4, wherein the AM layer and the HM layer exhibited a partially interpenetrative structure. This is because AM and HM have the same EPDM matrix, which allows them to coalesce with each other in the hot-pressing process. Such interpenetrative structure enabled the formation of strong interfaces and was beneficial for the resistance of hot gas erosion. Moreover, Figure S2 shows the layer thickness of the multilayer EPDM composites measured using the Image J software. Notably, all the multilayer EPDM composites exhibited uniform layer thickness except for (AM/HM)_80_. This might be due to the fact that the decrease in layer thickness led to a weakened layer strength, which tends to reduce the stability of layered flow during co-extrusion, and hence decreases layer regularity. 

### 3.2. Mechanical Properties

**Figure 7:**
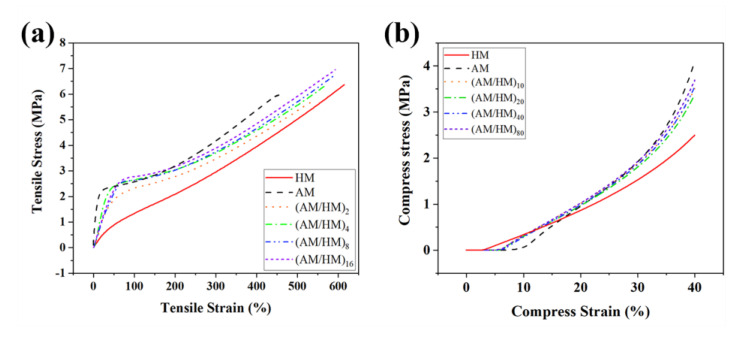
Tensile (**a**) and compressive (**b**) curves for the multilayer EPDM composite.

The mechanical properties of EPDM composites are of great significance for their practical use. In particular, the layered EPDM composite was composed of alternating AM and HM, wherein mechanical breakage often occurs at the interfaces under the action of external stress, which significantly affects the tensile properties. Compared with HM, AM showed a higher tensile modulus because the addition of hybrid fibers was beneficial for resisting the external stress in the low strain region. Moreover, AM showed poorer fracture strength and elongation at break than HM, because the poor adhesion of inert fibers with EPDM matrix resulted in weak interfaces, thus leading to inferior stress transfer. Figure 7a shows the influence of the layer numbers on the tensile properties of the multilayer EPDM composites. Notably, the multilayer EPDM composites showed combined properties of AM and HM. Especially, the fracture tensile strength of the multilayer EPDM composites was stronger than that of both AM and HM. The strength increased with the increase in the number of layers, indicating that the laminating structures enabled the enhancement of both robustness and toughness of EPDM composites. The reason for the higher tensile strength of EPDM laminates than the molded samples is that a high orientation occurs for the interspersed fibers due to the effects of shear flow and volumetric confinement during co-extrusion. Such anisotropic behavior became more efficient with the decrease in the layer thickness, which was beneficial for stress transfer of the EPDM composites.

Compression strength is another key factor, which influences the use of EPDM composites. The EPDM composites were compressed to 40% strain and repeated four times, and the last compressive stress–strain curves shown in Figure 7b were used to evaluate the compressive properties. The ablative EPDM showed a noticeable stress hysteresis compared with others, indicating that a greater permanent deformation occurs after cyclic compression. The increased hysteresis of AM was associated with the debonding between the fibers and the polymer chains, which hindered the strain recovery of the EPDM composites. Moreover, AM exhibited the highest compressive modulus because the robust fibrous skeleton after cyclic compressive loading was retained which facilitated the enhancement of the compression resistance. In contrast, HM showed the lowest compressive modulus which was attributed to the absence of rigid fibers. The compressive strength of the multilayer composites (AM/HM)_n_ was intermediate between the ablative EPDM and heat-insulated EPDM, and the increase in the layer number increased the compressive strength, which was due to the homogenization of the two different EPDM composites.

### 3.3. Thermal Stability of the Multilayer EPDM Composites

**Figure 8:**
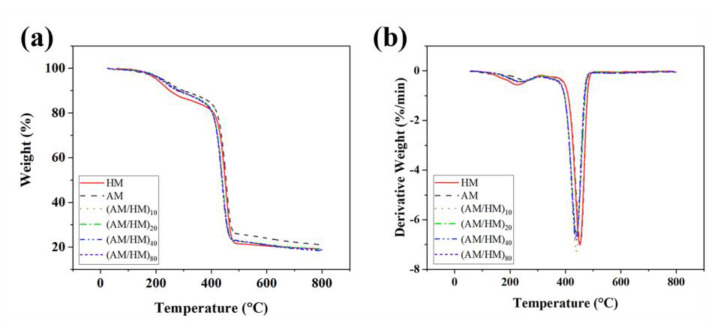
TGA (**a**) and DTG (**b**) plots of the multilayer EPDM composites under nitrogen atmosphere.

The thermal stability of the multilayer EPDM composites was studied by means of the thermogravimetry (TG)/derivative TG (DTG) methods. The thermal decomposition behavior of the compression molded EPDM and their alternating laminate composites under nitrogen conditions are shown in Figure 8, and the detailed data in the TG and DTG plots are tubulated in Table S2. Regardless of the compression molded heat-insulated EPDM, all the samples exhibit a three-stage degradation process. The mass loss below 300 °C was attributed to the removal of volatiles arising from the moisture loss, and the thermal degradation of the sulfamic moieties, accelerators and activators. The sharp main DTG peaks centered at approximately 455 °C are associated with the degradation of the EPDM matrix. Several indistinctive humps were observed at the temperature range of 500–800 °C, which are associated with the degradation process of AF. Notably, the multilayer EPDM composites showed lower decomposition temperatures than those of the compression molded samples because laminating structures enabled the planar orientation and densification of fillers, which was beneficial for forming heat conductive networks, thus promoting the decomposition of the EPDM matrix. The compression molded ablative EPDM showed the highest decomposition temperature and the largest equilibrium residual mass, which can be attributed to the presence of the largest proportion of CFs endowing the composites with superior thermal stability.

### 3.4. Thermal Conductivity of EPDM Composites

**Figure 9:**
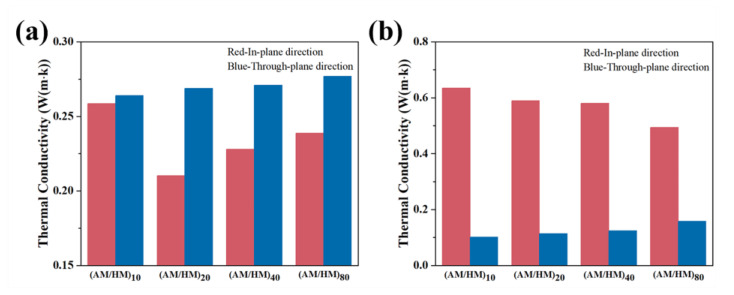
The in-plane and through-plane thermal conductivity of AH layer (**a**) and HM layer (**b**).

The thermal conductivity plays an important role in the heat transfer rate from the external environment to the interior matrix, which reflects the efficiency of thermal insulation of materials. Herein, the in-plane and through-plane thermal conductivities of the laminate (AM/HM)_n_ were tested at room temperature to assess their heat transfer rates. The ablative EPDM and heat-insulated EPDM in the laminate composite showed significant anisotropic thermal conductivities in the through-plane and in-plane directions, as shown in Figure 9. Specifically, the thermal conductivity of the ablative EPDM in the laminate composites showed much lower in-plane thermal conductivity than that in the through-plane direction. Additionally, the in-plane thermal conductivity of ablative EPDM was lower than the compression molded counterpart. This is because AFs with a very low thermal conductivity showed planar orientation along the extrusion direction, which was beneficial for decreasing the heat flux in the in-plane direction. The thermal conductivity increased with the decrease in the layer thickness, which could be attributed to the competition between the orientation of AFs and the densification of the high thermal conductive CFs. 

The thermal conductivity in the through-plane direction showed slightly higher values compared with the compression molded AM, which was attributed to the densification of CFs networks, resulting in a high thermal conductivity. Oppositely, the HM in the laminate composite showed significantly higher in-plane thermal conductivity compared with that in the through-plane direction, and higher than that of the molded HM. This anisotropic behavior might be due to the fact that the multistage shear forces during co-extrusion initiated planar orientation and densification of fillers, and the connection of the adjacent fillers in-plane enabled the formation of conductive pathways, thus enhancing the planar heat diffusion. In contrast, the concentration of thermal conductive fillers perpendicular to the extrusion direction was diluted, and the heat conductive pathway cannot be formed, which hindered the heat diffusion in the through-plane direction. 

The comprehensive thermal conductivity of the multilayer EPDM composites influenced by the in plane and through plane was measured through a hot-disk method, as shown in Table 1, which allowed us to investigate the thermal insulation of the multilayer composite. Results showed that the thermal conductivities of the multilayer EPDM composites were in the range of the molded heat-insulated EPDM and ablative EPDM. Specifically, the laminate composite (AM/HM)_40_ showed the lowest thermal conductivity, which was even lower than the molded HM. This is because the molded HM has a porous medium and its thermal conductivity (0.19 W/(m·k)) is much lower than that of the molded AM (0.28 W/(m·k)). Hence, the alternating distribution of HM acted as barrier to the heat flow, and the effect was more effective with the increase in the layer number. Owing to the alternating layered structure, the fiber networks formed in the ablative EPDM were interrupted and cannot be established throughout the laminating composite, which resulted in tortuous conductive pathways. Moreover, the in-plane thermal conductivity in HM was very effective, which facilitated the in-plane heat dissipation, with a consequent decrease in the heat diffusion in the through-plane direction. However, the barrier effect was decreased in the composite (AM/HM)_80_, which was attributed to the partial penetration of the high conductive fibers from the adjacent ablative EPDM. Overall, such anisotropic multilayer EPDM composites suggested great potential in the fabrication of multifunctional EPDM composites featuring both excellent ablation resistance and thermal insulation. 

### 3.5. Ablative Resistance of Multilayer EPDM Composites 

The excellent ablation resistance and thermal insulation are two important factors affecting the practical use of EPDM composites in the aerospace sectors. Herein, the ablative properties of EPDM composites were measured using the oxyacetylene torch, the ablation results, such as linear ablation rate and mass ablation rate, were calculated to evaluate the ablation properties. Additionally, the maximum back-face temperature (T_b_) in the oxyacetylene tests was monitored to assess the thermal insulation.

**Figure 10:**
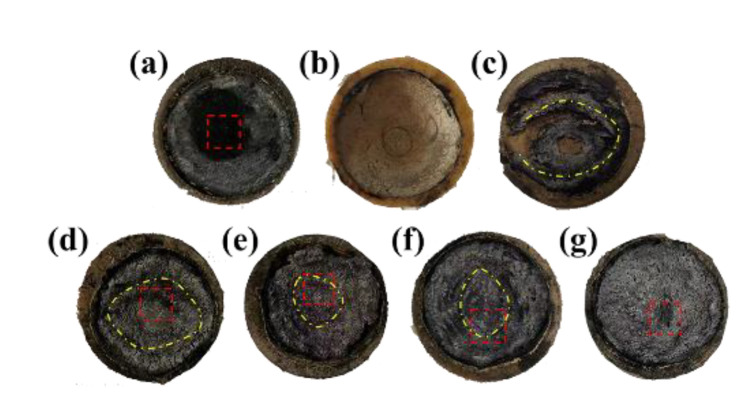
Macroscopic appearance of ablated EPDM composites. (Molded AM (**a**); Molded HM (**b**); (AM/HM)_6_ (**c**); (AM/HM)_10_ (**d**); (AM/HM)_20_ (**e**); (AM/HM)_40_ (**f**); (AM/HM)_80_ (**g**)).

**Figure 11:**
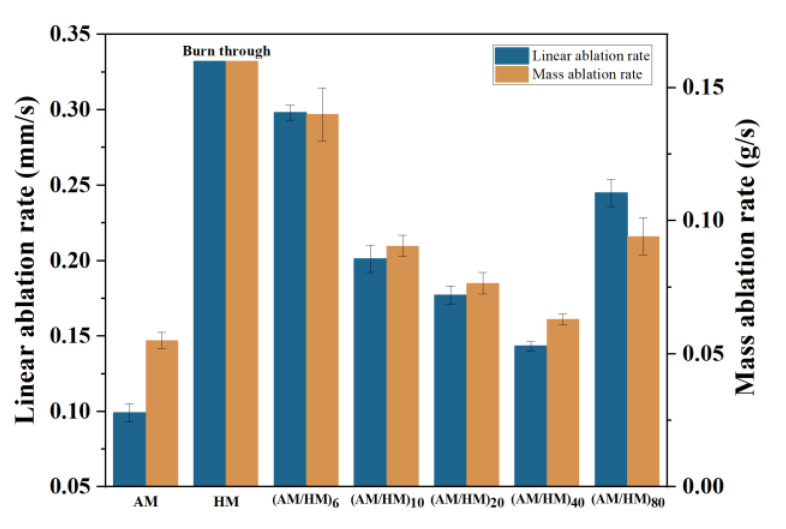
Variation in linear ablation rate (*R_l_*) and mass ablation rate (*R_c_*) as functions of layer numbers.

The molded HM was burnt through without the existence of the char layer (see Figure 10), which indicated a very poor resistance to the heat flux erosion. In contrast, the compression molded AM showed an intact char layer (see Figure 11), suggesting that the addition of fibers was beneficial for improving the char adhesion on the virgin materials. Moreover, the strengthening effect of fibers endowed AM with good resistance to heat erosion, resulting in the lowest linear ablation rate and mass ablation rate. Since the conventional compression molded AM and HM exhibited very different properties in terms of the ablation and thermal conductivity, the combination of them represents potential synergetic efficiencies, ensuring simultaneous satisfied ablation and insulation performances. 

**Figure 12:**
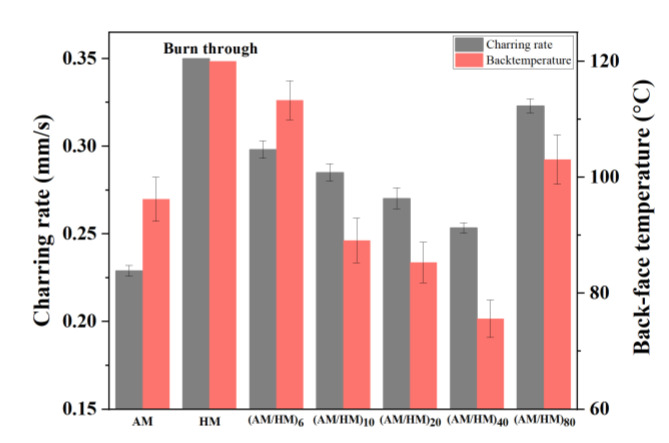
The *R_c_* and back-temperature of the (AM/HM)_n_.

The macroscopic appearance of ablated (AM/HM)_6_ showed alternating char layers corresponding to the carbonized products of AM and HM, respectively. Notably, the char layers were partially peeled off and the EPDM matrix was exposed, this is because the HM composite having very poor ablation resistance underwent a fast decomposition and burnt off during ablation. The removal of the adjacent layers allowed the acceleration of the transference of oxyacetylene flame, thus strengthening the hot gas erosion to the ablative EPDM, resulting in the increase in the ablation rates. However, the integrity of the char residues was improved with the increase in the layer number, which was beneficial for the enhancement of ablation resistance. In particular, compared with (AM/HM)_6_, the linear ablation rate of (AM/HM)_40_ was reduced by more than 50%, which was attributed to the planar orientation and densification of fibers at a high layer number. Such anisotropic behavior enabled the formation of denser ablative networks in the ablation surface, which was conducive to enhance the ablative resistance. Moreover, (AM/HM)_40_ showed the lowest maximum back-face temperature as shown in Figure 12, this is because the alternating lamination of AM and HM leads to an interruption of the fibrous networks, which contributes to the decrease in heat diffusion in the through-plane direction. However, it should be noted that (AM/HM)_80_ showed increased ablation rates and the back-face temperature than that of (AM/HM)_40_. This might be due to the fact that (AM/HM)_80_ with a lower char layer thickness had a poor strength, which made the char layers become small debris and burn off in the oxyacetylene flame. 

A partial penetration of the adjacent layers occurs under the action of multistage stretching during co-extrusion. Especially, when the layer thickness is lower than the length of the CFs, the CFs can penetrate efficiently through the adjacent two layers, resulting in the formation of conductive pathways, thus facilitating the decomposition of the pyrolysis of the inner matrix, consequently increasing the both of the ablation rates and back-face temperature. Hence, a proper laminating structure allows a combination of varied functional layers, which enabled the simultaneous enhancement of ablation resistance and thermal insulation. 

### 3.6. Morphological Analysis of the Char Layer

**Figure 13:**
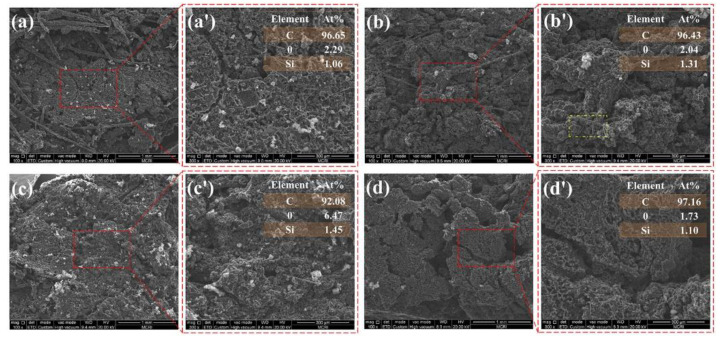
The SEM images of char layer ablation surface. ((AM/HM)_10_: (**a**); (AM/HM)_20_: (**b**); (AM/HM)_40_: (**c**); (AM/HM)_80_: (**d**)). (**a′**,**b′**,**c′**,**d′**) are the partial magnification of (**a**,**b**,**c**,**d**), respectively.

The ablative resistance and thermal insulation are associated with the structure of the char layer. Herein, the morphological analysis of the char layer after oxyacetylene ablation was performed by SEM to study the influence of laminate structure on the ablative and thermal insulative properties. The microstructures of the surface char layers are shown in Figure 13. The char layer of the EPDM laminate composite displayed a heterogeneous ablation surface, composed of loose carbonized fibrous skeleton and compact planar sectors. Such fragmental structure was unfavorable for resisting the long-time erosion of oxyacetylene flame, resulting in a high ablation and charring rate. The ablation surface became more intact and showed denser char residues with the increase in the number of layers, as shown in Figure 13b,c. This is because further multiplying the layer enabled simultaneous planar orientation and densification of the fibers in the ablation surface. Such anisotropic distribution was beneficial for strengthening the material’s mechanical properties, which endows the ablated char layer with improved compactness and strength. The compact char layer can restrict the diffusion of oxygen-rich gas and inhibits the thermal-chemical ablation of the interior matrix, resulting in decreasing ablation rates. Additionally, the compact char layer plays an important role to restrict the convective heat transfer and thermal radiation from the high temperature environment, which was responsible for the decrease in the back-face temperature. However, further increasing the layer number resulted in a loose and fragmented ablation surface as shown in Figure 13d, which indicated that the strength of the char layer was weakened with a further decrease in layer thickness. Additionally, this result can also explain the noticeable increase in the ablation rates and the back-face temperature of the laminate EPDM composites with a layer thickness of 0.125 mm.

**Figure 14:**
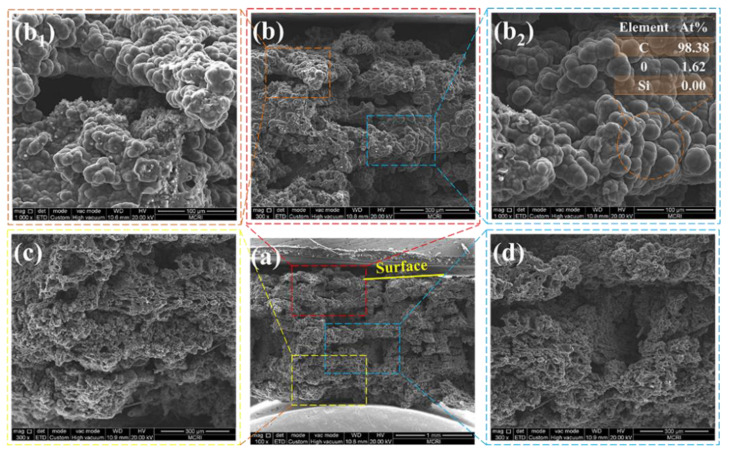
The SEM micrographs of the cross-section of the char layer of (AM/HM)_10_ (**a**). (upper (**b**,**b_1_**,**b_2_**), middle (**d**) and bottom (**c**) of char layer).

**Figure 15:**
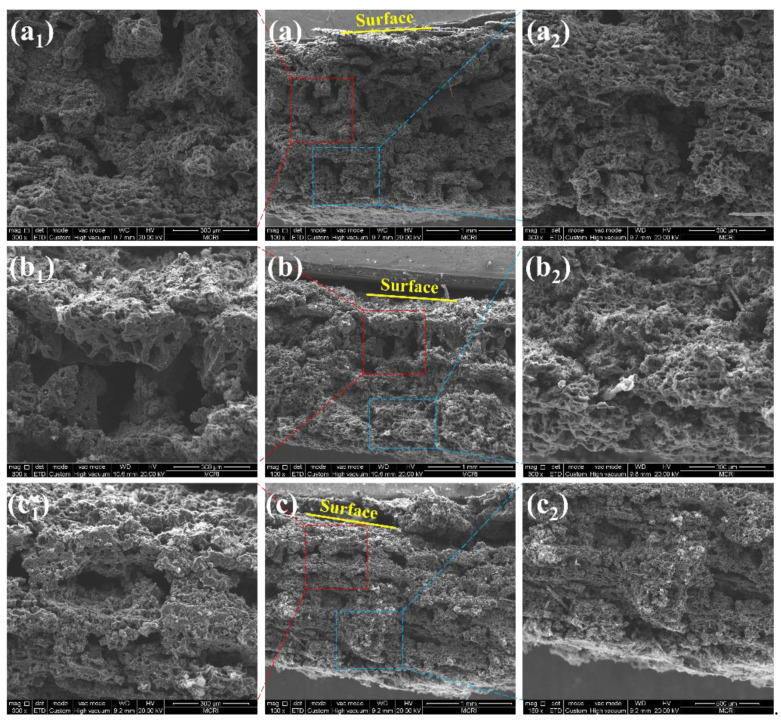
The SEM micrographs of the cross-section of the char layer. (AM/HM)_20_: (**a**); (AM/HM)_40_: (**b**); (AM/HM)_80_: (**c**). (**a_1_**,**a_2_**,**b_1_**,**b_2_**,**c_1_**,**c_2_**) are the partial magnification of (**a**,**b**,**c**), respectively.

The cross-sectional morphologies of the char layer yielded after ablation were also observed by SEM, as shown in Figure 14 and Figure 15. The char layer showed evidently alternating compact and loose structures, arising from the carbonization of the ablative EPDM and heat-insulated EPDM, respectively. The loose char layers displayed fragmented residual chars separated with numerous cavities because the strength of the char residues is very poor due to the absence of fibers in the heat-insulated EPDM, which facilitates the broken and debonding of chars during ablation. The results of the EDS analysis of the char residues highlighted in Figure 13 indicate that carbon-rich areas were formed in the upper parts of cavities. This is because the compact char layers can prevent pyrolysis gas from dissipating through plane. Especially, the ablative EPDM layers became denser after multistage co-extrusion, endowing EPDM composites with alternating barriers, which facilitated the deposition of pyrolysis products and densification of the char layers. The alternating compact and loose char layers play an important role for simultaneously improving the ablation resistance and thermal insulation. This is because the compact char layers featuring higher structural strength can resist the erosion of high-speed gas. In addition, the loose structure resulted in thermal barriers inside the char layer, which restrict the heat-transfer from the high temperature gas into the interior, thus benefiting the ablation resistance and thermal insulation of EPDM composites. 

## 4. Conclusions

EPDM multilayer composites consisting of an alternating ablative EPDM (AM) layer and heat-insulated EPDM (HM) layer were prepared by layer multiplying co-extrusion with the following conclusions.

1. Compared to conventional prepared ablative EPDM composites, the multilayer composites (AM/HM)_n_ showed improved thermal insulation and mechanical properties. The appearance of HM layers could separate the continuous conductive network in the through-plane direction resulting in the increase in the propagation path of heat diffusion.
2. The multilayer EPDM composites showed greater tensile strength and elongation at break compared to those of conventionally prepared AM and HM. The shear force in co-extrusion could improve the dispersion and induce the orientation of fibrous reinforcements along the flow direction, contributing to bearing the external stress.
3. EPDM composites with 40 alternating layers (AM/HM)_40_ demonstrated the most balanced properties in terms of the ablation, thermal insulation and mechanical strength.

Hence, tailoring the optimal multilayer architecture provides a great potential pathway to fabricate polymeric ablative materials with more outstanding comprehensive performance. 

## Figures and Tables

**Table 1:** Layer thickness and thermal conductivity of the (AM/HM)_n_.

Samples	Layer Thickness (mm)	Thermal Conductivity (W/(m·k))
HM	10	0.19
AM	10	0.28
(AM/HM)_6_	1.667	0.25
(AM/HM)_10_	1.000	0.2239
(AM/HM)_20_	0.500	0.2089
(AM/HM)_40_	0.250	0.1825
(AM/HM)_80_	0.125	0.2193

## Data Availability

Data from this study are available upon request from the corresponding authors.

## Supplementary Materials

The following supporting information can be downloaded at: https://www.mdpi.com/article/10.3390/polym14081570/s1, Figure S1: The SEM images of (a) microspheres, and (b) heat-insulated EPDM; Figure S2: The thickness of the multilayer EPDM composites with different layers, (a,a′) (AM/HM)_10_; (b,b′) (AM/HM)_20_; (c,c′) (AM/HM)_40_; (d,d′) (AM/HM)_80_; the AM component represented by brown, and HM component represented by blue.; Table S1: Formulation of EPDM materials; Table S2: Data of TG/DTG of the EPDM composites.

## Abbreviations

EPDM	Ethylene–propylene–diene monomer
SRM	Solid rocket motor
AM	Ablative EPDM
HM	Heat-insulated EPDM
PAMs	Polymeric ablation materials
LMEs	Layer multiplying elements
AF	Aramid fiber
CF	Carbon fiber
D	Diphenylguanidine
DM	Dibenzothiazole disulfide
EZ	Zinc diethyldithiocarbamate
(AM/HM)_n_	Multilayered EPDM composites with *n* layers
TGA	Thermogravimetric analysis
*R_l_*	Linear ablation rate
*R_c_*	Charring rate
*R_m_*	Mass ablation rate
T_b_	Back-face temperature
TG	Thermogravimetry
DTG	Derivative TG
