# Supplementary material for: Enhancing Thermal Insulation of EPDM Ablators via Constructing Alternating Planar Architectures
Source: Polymers (Basel). 2022 Apr 12;14(8):1570. doi: 10.3390/polym14081570 (PMC9028349; doi:10.3390/polym14081570)
Supplement: Supplementary file 1 [file polymers-14-01570-s001.zip › polymers-1666914-supplementary.pdf]

Supporting information

Enhancing thermal insulation of EPDM ablators via constructing alternating planar architecture

Hongjian Qu, Kun Hui, Le Wang, Cheng Bian, Hongyan Li, Yiwen Guan, Tao Luan, Ning Yan

Xi'an Modern Chemistry Research Institute, Shaanxi, China

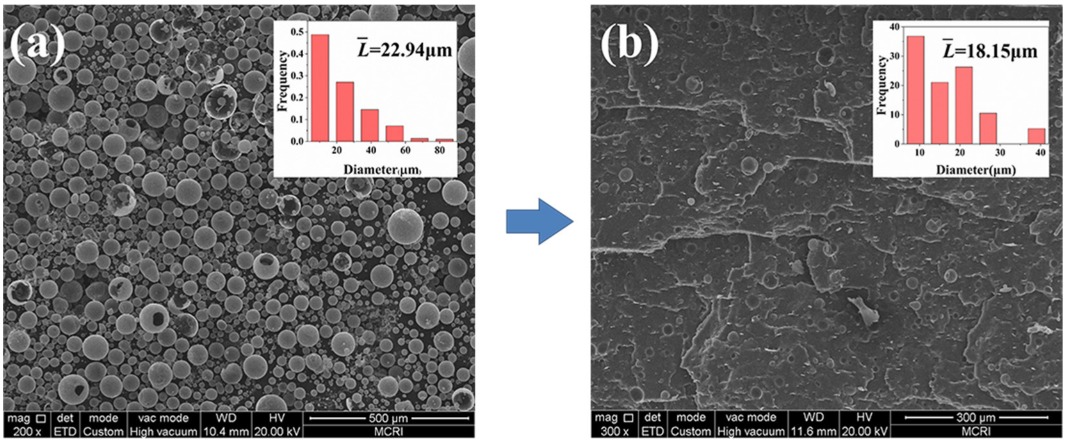

Figure S1. The SEM images of (**a**) microspheres, and (**b**) heat-insulated EPDM

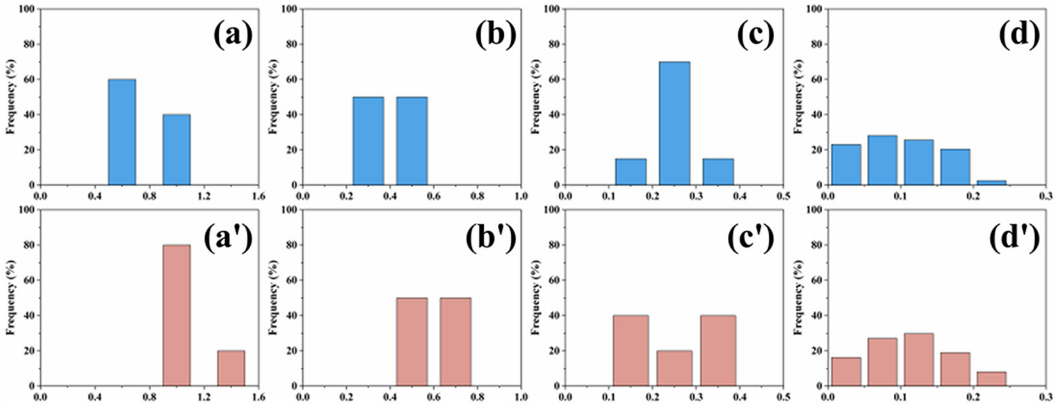

Figure S2. The thickness of the multilayer EPDM composites with different layers, (**a, a'**) (AM/HM)$_{10}$; (**b,b'**) (AM/HM)$_{20}$; (**c, c'**) (AM/HM)$_{40}$; (**d, d'**) (AM/HM)$_{80}$; the AM component represented by brown, and HM component represented by blue.

Table S1. Formulation of EPDM materials.

Samples	AM	HM
EPDM	100	100
Liquid EPDM	0	15
Boron Phenolic	20	0
DM	1.5	1.5
D	1	1
EZ	1	1
ZnO	3	3
SA	1	1
S	1.5	1.5
SiO_2	20	20
FL	2.5	0
CF	2.5	0
Borosilicate microspheres	0	10

Table S2. Data of TG/DTG of the EPDM composites.

Samples	Max decomposition temperature(℃)	Residual mass(%)
HM	452.30	19.238
AM	455.46	21.074
$(AM/HM)_{10}$	439.80	18.691
$(AM/HM)_{20}$	440.27	18.921
$(AM/HM)_{40}$	438.73	18.260
$(AM/HM)_{80}$	439.57	18.731
